# Supplementary material for: A Critical View of the Application of the APEX Software (Aqueous Photochemistry of Environmentally-Occurring Xenobiotics) to Predict Photoreaction Kinetics in Surface Freshwaters
Source: Molecules. 2019 Dec 18;25(1):9. doi: 10.3390/molecules25010009 (PMC7017383; doi:10.3390/molecules25010009)
Supplement: Supplementary file 1 [file molecules-25-00009-s001.zip › 3.2.4_gcc-4.4.0.zip!/3.2.4_gcc-4.4.0/3.2.4_gcc-4.4.0/doc/octave/html/interpreter/Object-Groups.html]

Object Groups - Untitled


Next: Graphics backends,
Previous: Callbacks,
Up: Advanced Plotting


---

#### 15.2.8 Object Groups

A number of Octave high level plot functions return groups of other
graphics objects or they return graphics objects that are have their
properties linked in such a way that changes to one of the properties
results in changes in the others. A graphic object that groups other
objects is an `hggroup`


— Function File: **hggroup** ()  
— Function File: **hggroup** (h)  
— Function File: **hggroup** (..., property, value, ...)  
> Create group object with parent h. If no parent is specified,
> the group is created in the current axes. Return the handle of the
> group object created.
>
> Multiple property-value pairs may be specified for the group, but they
> must appear in pairs.

For example a simple use of a `hggroup` might be

```
     x = 0:0.1:10;
     hg = hggroup ();
     plot (x, sin (x), "color", [1, 0, 0], "parent", hg);
     hold on
     plot (x, cos (x), "color", [0, 1, 0], "parent", hg);
     set (hg, "visible", "off");
```

which groups the two plots into a single object and controls their
visibility directly. The default properties of an `hggroup` are
the same as the set of common properties for the other graphics
objects. Additional properties can be added with the `addproperty`
function.

— Built-in Function: **addproperty** (name, h, type, [arg, ...])  
> Create a new property named name in graphics object h.
> type determines the type of the property to create. args
> usually contains the default value of the property, but additional
> arguments might be given, depending on the type of the property.
>
> The supported property types are:
>
> `string`: A string property. arg contains the default string value. `any`: An un-typed property. This kind of property can hold any octave value. args contains the default value. `radio`: A string property with a limited set of accepted values. The first argument must be a string with all accepted values separated by a vertical bar ('|'). The default value can be marked by enclosing it with a '{' '}' pair. The default value may also be given as an optional second string argument. `boolean`: A boolean property. This property type is equivalent to a radio property with "on|off" as accepted values. arg contains the default property value. `double`: A scalar double property. arg contains the default value. `handle`: A handle property. This kind of property holds the handle of a graphics object. arg contains the default handle value. When no default value is given, the property is initialized to the empty matrix. `data`: A data (matrix) property. arg contains the default data value. When no default value is given, the data is initialized to the empty matrix. `color`: A color property. arg contains the default color value. When no default color is given, the property is set to black. An optional second string argument may be given to specify an additional set of accepted string values (like a radio property).
>
> type may also be the concatenation of a core object type and
> a valid property name for that object type. The property created
> then has the same characteristics as the referenced property (type,
> possible values, hidden state...). This allows to clone an existing
> property into the graphics object h.
>
> Examples:
>
> ```
>           addproperty ("my_property", gcf, "string", "a string value");
>           addproperty ("my_radio", gcf, "radio", "val_1|val_2|{val_3}");
>           addproperty ("my_style", gcf, "linelinestyle", "--");
> ```

Once a property in added to an `hggroup`, it is not linked to any
other property of either the children of the group, or any other
graphics object. Add so to control the way in which this newly added
property is used, the `addlistener` function is used to define a
callback function that is executed when the property is altered.

— Built-in Function: **addlistener** (h, prop, fcn)  
> Register fcn as listener for the property prop of the graphics
> object h. Property listeners are executed (in order of registration)
> when the property is set. The new value is already available when the
> listeners are executed.
>
> prop must be a string naming a valid property in h.
>
> fcn can be a function handle, a string or a cell array whose first
> element is a function handle. If fcn is a function handle, the
> corresponding function should accept at least 2 arguments, that will be
> set to the object handle and the empty matrix respectively. If fcn
> is a string, it must be any valid octave expression. If fcn is a cell
> array, the first element must be a function handle with the same signature
> as described above. The next elements of the cell array are passed
> as additional arguments to the function.
>
> Example:
>
> ```
>           function my_listener (h, dummy, p1)
>             fprintf ("my_listener called with p1=%s\n", p1);
>           endfunction
>           
>           addlistener (gcf, "position", {@my_listener, "my string"})
> ```

— Built-in Function: **dellistener** (h, prop, fcn)  
> Remove the registration of fcn as a listener for the property
> prop of the graphics object h. The function fcn must
> be the same variable (not just the same value), as was passed to the
> original call to `addlistener`.
>
> If fcn is not defined then all listener functions of prop
> are removed.
>
> Example:
>
> ```
>           function my_listener (h, dummy, p1)
>             fprintf ("my_listener called with p1=%s\n", p1);
>           endfunction
>           
>           c = {@my_listener, "my string"};
>           addlistener (gcf, "position", c);
>           dellistener (gcf, "position", c);
> ```

An example of the use of these two functions might be

```
     x = 0:0.1:10;
     hg = hggroup ();
     h = plot (x, sin (x), "color", [1, 0, 0], "parent", hg);
     addproperty ("linestyle", hg, "linelinestyle", get (h, "linestyle"));
     addlistener (hg, "linestyle", @update_props);
     hold on
     plot (x, cos (x), "color", [0, 1, 0], "parent", hg);
     
     function update_props (h, d)
       set (get (h, "children"), "linestyle", get (h, "linestyle"));
     endfunction
```

that adds a `linestyle` property to the `hggroup` and
propagating any changes its value to the children of the group. The
`linkprop` function can be used to simplify the above to be

```
     x = 0:0.1:10;
     hg = hggroup ();
     h1 = plot (x, sin (x), "color", [1, 0, 0], "parent", hg);
     addproperty ("linestyle", hg, "linelinestyle", get (h, "linestyle"));
     hold on
     h2 = plot (x, cos (x), "color", [0, 1, 0], "parent", hg);
     hlink = linkprop ([hg, h1, h2], "color");
```

— Function File: hlink = **linkprop** (h, prop)  
> Links graphics object properties, such that a change in one is
> propagated to the others. The properties to link are given as a
> string of cell string array by prop and the objects containing
> these properties by the handle array h.
>
> An example of the use of linkprops is
>
> ```
>           x = 0:0.1:10;
>           subplot (1, 2, 1);
>           h1 = plot (x, sin (x));
>           subplot (1, 2, 2);
>           h2 = plot (x, cos (x));
>           hlink = linkprop ([h1, h2], {"color","linestyle"});
>           set (h1, "color", "green");
>           set (h2, "linestyle", "--");
> ```

These capabilities are used in a number of basic graphics objects.
The `hggroup` objects created by the functions of Octave contain
one or more graphics object and are used to:

- group together multiple graphics objects,- create linked properties between different graphics objects, and- to hide the nominal user data, from the actual data of the objects.

For example the `stem` function creates a stem series where each
`hggroup` of the stem series contains two line objects representing
the body and head of the stem. The `ydata` property of the
`hggroup` of the stem series represents the head of the stem,
whereas the body of the stem is between the baseline and this value. For
example

```
     h = stem (1:4)
     get (h, "xdata")
     => [  1   2   3   4]'
     get (get (h, "children")(1), "xdata")
     => [  1   1 NaN   2   2 NaN   3   3 NaN   4   4 NaN]'
```

shows the difference between the `xdata` of the `hggroup`
of a stem series object and the underlying line.

The basic properties of such group objects is that they consist of one
or more linked `hggroup`, and that changes in certain properties of
these groups are propagated to other members of the group. Whereas,
certain properties of the members of the group only apply to the current
member.

In addition the members of the group can also be linked to other
graphics objects through callback functions. For example the baseline of
the `bar` or `stem` functions is a line object, whose length
and position are automatically adjusted, based on changes to the
corresponding hggroup elements.

- Data sources in object groups- Area series- Bar series- Contour groups- Error bar series- Line series- Quiver group- Scatter group- Stair group- Stem Series- Surface group
